# Urban fires as public health emergencies: lessons from the Gul Plaza disaster in Karachi

**DOI:** 10.1097/MS9.0000000000005059

**Published:** 2026-04-27

**Authors:** Syed Ahtisham Halim, Syed Muhammad Safiullah, Kamil Ahmad Kamil, Mohammad Naeem, Samsoor Sultanzai, Humayoon Aziz Zakhailwal, Jawad Shirzad

**Affiliations:** a Department of Medicine, Jinnah Sindh Medical University, Karachi, Pakistan; b Internal Medicine Department, Mirwais Regional Hospital, Kandahar, Afghanistan; c Department of Surgery, Jinnah Postgraduate Medical Centre, Karachi, Pakistan; d Department of Public Health, Kandahar University, Kandahar, Afghanistan

*Dear Editor,*

This letter to the editor adheres to the TITAN guideline on the need for transparency in AI use in healthcare^[^1^]^. The recent fire at the Gul Plaza located in Karachi resulted in the deaths of 79 individuals. More than 20 people were injured^[^2^]^, while 49 were reported missing during rescue operations^[^3^]^. In several cases, only body parts were recovered, while DNA testing had to be conducted to identify some victims^[^4^]^. it is one of the most devastating urban fire-related disasters that has occurred in Pakistan either recently or during the last decade. The preliminary report indicates that fire was directly attributed to a lighter; however, the overwhelming majority of the damage occurred due to systemic failures of the fire suppression systems that solely relied upon the use of water for suppression, without utilizing foam-type or sprinkler systems. Moreover, the enforcement of the building codes also played a role in the extent of the damage caused by the fire^[^5,6^]^.

The recent history of Pakistan has witnessed some major catastrophic tragedies. The fire incident at the Baldia Town garment factory in Karachi resulted in the deaths of more than 250 workers because the exits were closed and there was no proper way of rescue response. In the recent past, the Gadani shipyard fire resulted in the deaths of many workers because of the explosions caused by the tankers. In the year 2025, Karachi witnessed more than 2400 fire incidents^[^7^]^. Such tragedies have also occurred in Dhaka’s garment industry and in the hospital industry in India^[^8^]^.

According to global best practices, the implementation of fire safety infrastructure is associated with reduced mortality rates. For example, the UNECE International Fire Safety Standards (2020) encourage the use of fire suppression systems such as sprinklers, compartmentation, and smoke control^[^9^]^. Also, the Global Plan for a Decade of Action for Fire Safety^[^10^]^ encourages the use of advancements in firefighting technology and greater public awareness of fire safety. For example, both Tokyo and London have mitigated the effects of large-scale and destructive fire events through the use of foam-based suppression systems, water mist technologies, and the implementation of stringent fire safety building codes. The Grenfell Tower fire in London in 2017 resulted in new regulatory frameworks related to fire safety including cladding and removing occupants from buildings in a fire emergency^[^11,12^]^.

Fire disasters in urban areas are predictable from a public health perspective and can be prevented by implementing appropriate recommendations that address both vulnerable urban-dwelling populations and their need for fire protection. Recommendations for South Asian countries to help reduce the repetition of fire tragedies include upgrading fire-fighting equipment, enforcing building standards, regulating fire registries, and increasing public knowledge of fire safety and first aid. School-based disaster risk reduction programmes and workplace training, as promoted by the International Federation of Red Cross and Red Crescent Societies^[^13^]^, can empower citizens to provide immediate assistance before professional responders arrive.

The Gul Plaza tragedy must serve as a turning point. Integrating fire safety into urban health policy will reduce mortality and morbidity, strengthen resilience, and align South Asia with global standards.

## Data Availability

All data used are from publicly available published sources.
